# Geriatric patients with multidrug resistant organisms in sepsis with community acquired pneumonia, pedic diabeticulcer, decubitus ulcer stage III, diabetic kidney disease stage V, urinary tract infection and anemia : case report

**DOI:** 10.1017/ash.2025.98

**Published:** 2025-09-03

**Authors:** Roro Flaviana Bayu Astiyani, Dhani Redhono Harioputro, R. Satriyo Budhi Susilo, Tatar Sumandja, Arifin, Evi Nurhayatun

**Affiliations:** 1 Fellowship in Infectious and Tropical Disease Division Medical Faculty UNS, dr. Moewardi Hospital, Surakarta, Indonesia; 2 Infectious and Tropical Disease Division Medical Faculty UNS dr. Moewardi Hospital, Surakarta, Indonesia

**Keywords:** MDRO, Sepsis, Antibiotics, Mortality

## Abstract

**Case Presentation:** A 68 year old man. Hospitalized with decreased consciousness. Experienced severe shortness of breath 3 days before entering the hospital. The patient also had wounds on his right and left legs since 1 month ago. But then became more widespread. The patient has kidney failure and routinely undergoes hemodialysis. The patient had diabetes since 6 years ago. Laboratory: Hemoglobin 7.5 Leukocytes 17.8 Netrophils 91.70 Lymphocytes 4.20 Albumin 2.2 Creatinine 2.5 Ureum 61 Artery 2.30, urine bacteria+++. Pus culture results: Enterobacter cloacae with the antibiotic meropenem. Sputum culture results Klebsiella pneumoniae ss. Pneumoniae with amikacin. After 1 week pus culture results: Pseudomonas aeruginosa with amikacin. Blood culture results: Staphylococcus epidermidis suggested vancomycin. The patient underwent debriment in the operating room. However, the condition did not improve. **Discussion:** This patient experienced sepsis with MDRO. Apart from geriatric age, the patient also has diabetes with complications of kidney failure. This worsens the patient’s immune system. So the patient’s diabetic ulcers and decubitus ulcers worsened with the results of cultures with various antibiotic-resistant multiorganisms. And also the respiratory infections increase the risk of mortality. **Conclusion :** MDRO is a risk factor for inappropriate antibiotic therapy, which is undoubtedly associated with increased mortality.

**Figure :**
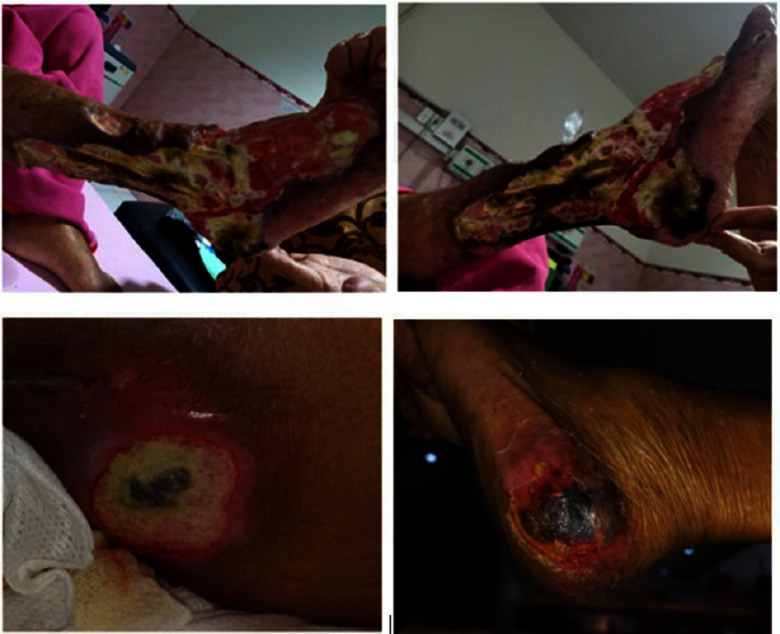
Diabetic ulcers and decubitus ulcers in patient

Diabetic ulcers and decubitus ulcers in patient

